# Facility-Level Access Drives Disparities in Influenza and Pneumococcal Vaccination in Long-Term Care Facilities in Southern Poland

**DOI:** 10.3390/idr18030045

**Published:** 2026-05-12

**Authors:** Zofia Gniadek, Estera Jachowicz-Matczak, Cezary Kapturkiewicz, Izabella Bylica, Dorota Romaniszyn, Jadwiga Wójkowska-Mach

**Affiliations:** 1 Department of Infection Control and Mycology, Faculty of Medicine, Jagiellonian University Medical College, 31-121 Kraków, Poland; zofia.gniadek@alumni.uj.edu.pl (Z.G.); estera.jachowicz-matczak@uj.edu.pl (E.J.-M.); ckapturkiewicz@su.krakow.pl (C.K.); izabella.owsianka@uj.edu.pl (I.B.); d.romaniszyn@uj.edu.pl (D.R.); 2 Faculty of Medicine and Health, University of Applied Sciences in Tarnow, 33-100 Tarnów, Poland

**Keywords:** influenza vaccination, pneumococcal vaccination, long-term care facilities, older adults

## Abstract

Background: Vaccinations prevent severe respiratory infections in older adults, yet uptake in Polish long-term care facilities (LTCFs) remains poorly characterized. We assessed influenza and pneumococcal vaccination coverage and factors associated with uptake, including the influence of local government financing. Methods: In this prospective observational study (January–June 2022), residents aged ≥65 years from eight LTCFs in southern Poland (four public, four private) were evaluated. Clinical data and geriatric assessments (Barthel Index, ADL, FRAIL-NH) were obtained from medical records and questionnaires. Comparative analyses were limited to residents living in facilities where vaccination activities were implemented and for whom complete data were available. Results: Overall, 429 residents were assessed: 136 (31.7%) received influenza vaccination and 77 (17.9%) received pneumococcal vaccination. Three of the eight LTCFs administered neither influenza nor pneumococcal vaccines, highlighting a facility-level access gap. For individual-level comparisons, 260 residents with complete data from LTCFs offering vaccination were analyzed (245 for pneumococcal outcomes). Influenza vaccination was not associated with most comorbidities or functional measures, but was more common among residents with dementia. Pneumococcal vaccine recipients were younger, had better functional status, and exhibited a lower burden of comorbidities than unvaccinated residents, suggesting preferential vaccination of fitter individuals. Municipality-level data showed low uptake of publicly funded pneumococcal programs (6.1% in Kraków; 3.6% in Wilkowice). Conclusions: Vaccination coverage among LTCF residents was low and strongly influenced by structural access at the facility level. Simplifying costs, reducing out-of-pocket costs and addressing potential age-related biases are essential to improving equitable immunization in Polish LTCFs.

## 1. Introduction

Vaccination is one of the greatest achievements of modern medicine and has undoubtedly contributed to a substantial reduction in infectious diseases [1]. The introduction of vaccines has been particularly important in decreasing infant and child mortality [2]. Despite widespread promotion of childhood vaccinations, older adults often receive less attention. Due to factors like immunosenescence and comorbidities, they are at higher risk of severe disease or death from infections. Vaccination, especially for influenza and pneumococcal diseases, is highly recommended for this group.

Flu vaccines, including high-dose or adjuvanted formulations, utilize quadrivalent (four-strain, used in Poland since 2017) technology to provide protection against multiple circulating influenza strains, boosting immune response. Pneumococcal vaccines, such as the 23-valent polysaccharide vaccine (PPSV23), not available in Poland as of 2024, along with the 20-valent (PCV-20), 13-valent (PCV13) and 10-valent (PCV10) conjugate vaccines, offer protection against various pneumococcus strains. These advanced vaccine technologies provide broader coverage and stronger immunity, reducing the risk of severe illness, hospitalization, and complications in older adults. In Poland, only influenza and COVID-19 vaccines are free for those over 65. Additionally, certain rare medical conditions—such as post-organ or bone marrow transplantation—qualify individuals for free pneumococcal vaccination. Some local governments also implement municipal vaccination programs that provide free access to selected vaccines for their residents.

Poland is experiencing rapid population aging, which requires increased attention to the challenges faced by older adults, particularly those that are preventable. During our prospective research on infections among residents of long-term care facilities (LTCFs) [3], we were struck by the low rates of pneumococcal and influenza vaccination among residents. This observation prompted us to examine the availability of, and factors influencing, influenza and pneumococcal vaccination among LTCF residents in southern Poland, including the role of local government in financing additional vaccination programs.

In Poland, the availability and uptake of adult vaccinations remain insufficiently characterized due to a lack of comprehensive national data. While selected immunization programs exist, particularly for influenza and COVID-19, there is no consistent, centralized system for monitoring vaccination coverage among adults, especially older populations. This data gap makes it difficult to accurately measure vaccine access, identify disparities between regions or population groups, and evaluate the effectiveness of public health interventions. Consequently, the true level of adult vaccination coverage—including vaccines against pneumococci—remains uncertain, limiting evidence-based decision-making and policy development in this area.

## 2. Methods

An observational prospective study was conducted among older adults aged ≥65 years, recruited from the eight long-term care facilities in southern Poland. Invitations to participate in the study were sent to all private and public LTCFs in southern Poland, using the public database at https://rpwdl.ezdrowie.gov.pl/RPM/ (accessed on 29 April 2026). The study was conducted from January to June, and included only the eight facilities that agreed to participate. Trained medical personnel reviewed electronic and paper records to analyze residents’ chronic diseases, medications, and medical issues. In addition, the following scales were used to assess the functional status, independence, and general health condition of the older adults included in the study [4]:

- The activities of daily living (ADL) scale (range: 0–6) assesses the ability to perform six basic activities of daily living. Higher scores indicate greater independence in performing daily activities.
- The FRAIL-NH scale (range: 0–14) is a screening tool developed to assess frailty syndrome among nursing home residents. Higher scores reflect a greater degree of frailty.
- The Barthel Index (range: 0–100) evaluates ten variables related to activities of daily living and mobility. Higher scores indicate greater independence, while lower scores reflect a higher degree of dependence.

Residents aged ≥65 years from long-term care facilities (LTCFs) were eligible for inclusion in individual-level analyses if vaccination activities against influenza or pneumococci were implemented in the facility during the study period. Facilities in which no vaccinations were administered were excluded from comparative analyses, as residents in these settings lacked structural access to immunization, precluding meaningful assessment of individual-level determinants of vaccination uptake. This restriction was applied to ensure that comparisons between vaccinated and unvaccinated residents were conducted within LTCFs offering comparable access to vaccination, thereby minimizing confounding by facility-level organizational barriers. Residents with incomplete clinical or vaccination data were excluded. For pneumococcal vaccination analyses, cases with missing outcome-specific data were additionally excluded.

### Assessment of Local Vaccination Financing Programs

To gain a complete understanding of the local situation, data were collected from the local governments of the Małopolskie [4] and Śląskie [5] regions (where the studied LTCFs were located) on the financing of voluntary programs for vaccinations against pneumococci. In September 2024, the authors sent inquiries to both communes about the number of vaccinations administered in all people in 2022 which were covered under their vaccination financing programs, regardless of whether they were the subject of the study or not. To contextualize local pneumococcal vaccination financing programs, we additionally obtained municipality-level data for 2021–2022: the population aged ≥65 years was 4077 in Kraków and 2669 in Wilkowice, and pneumococcal vaccination was administered to 250 (6.1%) and 96 (3.6%) individuals, respectively.

Statistical analysis was performed using TIBCO STATISTICA software, ver. 13.3.721.0 (TIBCO Software Inc., Palo Alto, CA, USA), licensed to Jagiellonian University—Medical College. Absolute values were used to describe nominal variables, as well as median values with interquartile ranges (1Q and 3Q). In most cases, the data did not follow a normal distribution; therefore, the non-parametric Mann–Whitney U test was used for quantitative variables, and the chi-square test was used for dichotomous variables. While multivariate analysis was initially considered, it was ultimately omitted due to multiple data limitations: lack of complete data from every participant, multiple variables being co-dependent, and small group size for crucial variables, especially numerical. For those reasons, the model could be highly unstable, and the results of such analysis could be potentially misleading. In all analyses, the significance level was *p* < 0.05.

## 3. Results

The study was conducted in long-term care facilities (LTCFs) in southern Poland, including residential social care homes (DPS) and medical long-term care institutions (ZOL and ZPO). According to official voivodeship registers and National Health Fund data, the Małopolskie and Śląskie regions comprise 214 and 287 LTCFs, respectively (501 in total): eight facilities participated in the study (1.6% of all LTCFs in these regions) [6,7], four public LTCFs (n = 234 residents) and four private LTCFs (n = 195 residents).

**Figure 1:**
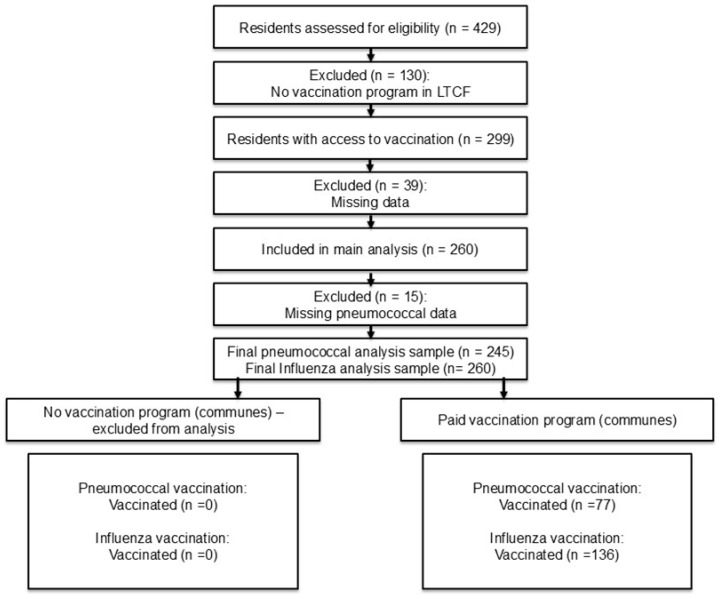
Participant flow and clinical data eligibility for the study according to access to influenza and pneumococcal vaccination at the facility level.

Overall, 429 residents were initially assessed. Three facilities reported no influenza or pneumococcal vaccination activities; therefore, 130 residents (30.3%) were excluded from comparative analyses because they lacked structural access to immunization. Among residents from facilities where vaccination activities were implemented, 39 were excluded due to incomplete clinical or vaccination data, resulting in a final analytical sample of 260 residents. For pneumococcal vaccination analyses, a further 15 residents were excluded due to missing outcome-specific data. This approach ensured that comparisons between vaccinated and unvaccinated residents were performed within settings offering comparable access to vaccinations (Figure 1). Questionnaire data indicated that 93% of surveyed LTCF residents had received COVID-19 vaccination (Table 1).

In the study population, after excluding facilities where no vaccinations were carried out, 136 (45.48% of all) residents were vaccinated against influenza and 77 (25.75%) against pneumococci, including 45 (15.05%) residents who were vaccinated against both influenza and pneumococci. Influenza vaccination was more common than pneumococcal vaccination, and receiving one vaccine significantly increased the likelihood of receiving the other (*p* < 0.001, Figure 1). 

Considering only the LTC units where vaccinations were performed, it was found that the clinical condition of the residents had no influence on the use of the influenza vaccine, except dementia (51.9% vs. 38.7%, *p* = 0.03), which increased the likelihood of vaccination. Pulmonary fibrosis (1.5% vs. 7.5%, *p* = 0.02) appeared to be a potential predictor of influenza vaccination.

Residents who received pneumococcal vaccination were younger (medians: 75 years (IQR 69.0–84.0) vs. 83 years (IQR 72.0–83.0), *p* = 0.001) and scored better on geriatric functional assessment scores (Barthel scale, ADL scale, and FRAIL-NH scale) compared to unvaccinated residents (Table 1).

Vaccinated residents were also less likely to suffer from frailty syndrome (32.4% vs. 51.8% in non-vaccinated residents, *p* = 0.005). They also had lower prevalence of atrial fibrillation (14.3% vs. 26.2%, *p* = 0.04), heart failure (25.0% vs. 48.8%, *p* < 0.001), depression (28.6% vs. 45.5%, *p* = 0.01) and osteoarthritis (37.7% vs. 63.7, *p* < 0.001). Vaccinated residents also had fewer chronic diseases overall (median; 5 [3.0–7.0] vs. 7; [5.0–9.0]; Table 1).

## 4. Discussion

Our findings reveal a two-level pattern of vaccination disparities. At the structural level, nearly one-third of the studied long-term care facilities had no vaccination activity at all, indicating systemic inequities in access. At the individual level, determinants of vaccination could only be examined among residents of facilities in which vaccination programs were implemented. In the studied LTCF resident population of southern Poland, only one-third of all residents were vaccinated against influenza, one-fifth against pneumococci and just 10% received both vaccines. Unexpectedly, pneumococcal vaccine recipients were younger and had fewer comorbidities, which likely reflects selection bias rather than being a true determinant of vaccination. Every year, influenza affects 5–15% of the global population. In Poland, during the epidemic season of 2022/2023, the incidence of influenza and influenza-like infections reached 8% [8]. Pneumococcal pneumonia is also a significant concern for the geriatric population in Poland. Between 2009 and 2020, there were 84,367 in-hospital deaths due to community-acquired pneumonia (CAP), with 0.5% attributed to Streptococcus pneumoniae, most of which occurred in patients aged 60 and older [9].

Flu vaccination coverage in Europe varies. In 2022, Denmark, Ireland, and the Netherlands had rates above 70%, while countries like Lithuania, Bulgaria, and Poland were below 10%. Among Poles aged 65 and over, only 8.6% received the flu vaccine in 2022, just slightly above Slovakia’s 5.6% [10]. Europe’s pneumococcal vaccination rates remain low, averaging 17.95% among at-risk groups [11].

Vaccination, as a primary prevention measure, is especially recommended for individuals with chronic illnesses, a higher risk of infection, and those residing in facilities to promote herd immunity. According to Naquin A et al., the most common conditions among patients hospitalized due to influenza included cardiovascular disease, hypertension, chronic metabolic disease, chronic lung disease, and neurological disorders [12]—yet, in our study, these groups were not more likely to be vaccinated against influenza.

Our team focused on exploring why some residents received vaccinations, primarily considering that certain diseases, which increase the risk of severe complications from infections, may influence vaccination decisions. We aimed to examine the role of specific diseases as key factors in vaccination uptake. Samel-Kowalik et al. observed a significant negative association between religious faith and attitudes towards influenza vaccination in Poland. Respondents who identified as non-religious were over four times more likely to have a positive attitude towards seasonal influenza vaccination compared to those with strong religious beliefs [13]. However, compared to the high percentage of patients who received the COVID-19 vaccine, the low uptake of other vaccinations may be attributed to a lack of adequate information about vaccinations and the diseases they prevent, particularly the risks to older adults. Unfortunately, our questionnaire did not address reasons for non-vaccination, as it was designed to gather broader health-related data and information on infection prevention, not to focus on vaccinations. The unexpectedly low vaccination rates were not anticipated, which explains why the questionnaire did not address this issue.

The most difficult finding to explain is that one-third of the examined LTCFs had no systemic support for vaccination—no vaccinations were administered at all. One hypothesis for the poor access to vaccinations among the studied population of residents in Polish LTCFs could be a lack of understanding of the importance of vaccinations in preventing infections among older adults.

It is concerning that in the studied population, approximately one-third of residents lived in facilities without any vaccination program. Unfortunately, data from the municipalities of Kraków and Wilkowice are also concerning—with only 5.1% of eligible older residents using free pneumococcal vaccination programs. The healthcare system in Poland does not adequately support primary infection prevention for older people, even when local governments are involved in vaccination reimbursement systems, as in Kraków and Wilkowice. For example, the Polish system for influenza vaccination is hindered by procedural difficulties. Recipients must first obtain a prescription, purchase the vaccine at a pharmacy (or receive it for free if they are older), and then visit a primary care physician for administration.

One of the biggest barriers to vaccination, despite its availability, is cost. Many older adults cannot afford vaccines, as they must prioritize essential expenses. When comparing the cost of vaccination to the potential expenses of treating a disease, especially for those requiring hospitalization, financing vaccinations appears more cost-effective. For example, a single dose of the pneumococcal vaccine (PCV-13) costs 280.12 zloty (65.65 EUR), while the median retirement pay in 2022 was 2452.94 zloty (574.9 EUR) [14]. In Poland, residents of long-term care facilities are responsible for covering the cost of their own medications, including vaccines. These expenses are not financed by the facilities themselves, nor are they typically included in the standard fees paid for accommodation and care. As a result, the financial burden of purchasing prescribed drugs and recommended vaccines often falls entirely on the residents, many of whom rely solely on limited retirement income. This likely contributes to the low vaccination rates observed among this population.

Cost is a major barrier to vaccination despite its availability. Many older adults must prioritize essential expenses, making vaccines less affordable. For example, a pneumococcal vaccine (PCV-13) costs 280.12 PLN, while the median pension in 2022 was 2452.94 PLN [14]. In Poland, residents of long-term care facilities must cover the cost of their own medications, including vaccines, which are not included in standard care fees. This financial burden may contribute to low vaccination rates in this population.

Vaccination is the most cost-effective way to reduce healthcare costs, morbidity, and mortality and should be strongly considered for all individuals without contraindications [15]. Despite vaccination programs in Kraków and Wilkowice, pneumococcal vaccination rates remain low, highlighting challenges in achieving high coverage. Even with free vaccines like the influenza vaccine, rates among older adults remain low.

This suggests that financial barriers are not the only obstacle. Misinformation, fear of side effects, and insufficient recommendations from healthcare providers also play a role. These challenges underscore the urgent need for effective strategies to promote vaccination among older adults, particularly in LTCFs, as they present significant health, social, and economic risks.

It is also worth noting that, despite both vaccinations being available free of charge for this population, a considerable difference in vaccination coverage persists. This disparity may be attributed to the greater public awareness and promotion of influenza vaccination, as well as the broader media attention that seasonal influenza typically receives compared to pneumococcal disease.

## 5. Conclusions

In the studied population of Polish LTCFs, receiving one vaccine increased the likelihood of receiving another. However, the overall vaccination rate for vaccines available free of charge remained very low, well below the expected level. Combating ageism and addressing the lack of awareness are crucial to improving vaccination rates in long-term care facilities. Ageism can lead to the undervaluation of older adults’ health needs, resulting in insufficient promotion of vaccination for this vulnerable group.

## 6. Limitations

Our study has several limitations. Limiting comparative analyses to LTCFs with implemented vaccination activities introduces facility-level selection and restricts generalizability; however, this approach was necessary to disentangle individual-level determinants of vaccination from structural barriers related to access. Estimates in selected clinical subgroups (e.g., pulmonary disease, liver cirrhosis, rheumatoid arthritis) were constrained by small sample sizes, limiting the precision of subgroup comparisons and the reliability of inferences. We did not collect data on participants’ reasons for non-vaccination (for specific vaccines or for the current year). This is because low vaccination prevalence was unexpectedly pronounced and so no questionnaire items addressing reasons for non-vaccination were included. Finally, although multivariable modeling was initially considered, it was not performed due to incomplete data for some participants, interdependence/collinearity among several covariates, and small numbers for key variables (especially numerical predictors). Under these conditions, multivariable models would likely be unstable and potentially misleading; therefore, we restricted the analyses to descriptive and univariable comparisons.

## Figures and Tables

**Table 1:** Coverage of influenza and pneumococcal vaccinations in the population of qualified residents of five LCTFs from the eight studied LCTFs.

Demographic and Clinical Characteristic of Residents	Influenza Vaccination			Pneumococcal Vaccination *		
	Yes (n = 136)	No (n = 124)	*p*-Value	Yes (n = 77)	No (n = 168)	*p*-Value
Age, Me (Q1–Q3)	81 (70.5–87.0)	83 (70.0–88.0)	0.594	75 (69.0–84.0)	83 (72.0–83.0)	0.001
Male, n (%)	56 (41.2)	42 (33.9)	0.225	35 (45.5)	52 (33.9)	0.084
BMI, Me (Q1–Q3)	26 (22.4–29.0)	25 (21.6–27.9)	0.082	26 (22.7–30.4)	25 (21.8–27.9)	0.120
Chronic illnesses, Me (Q1–Q3)	7 (4.0–9.0)	6 (4.0–9.0)	0.356	5 (3.0–7.0)	7 (5.0–9.0)	0.001
Number of drugs, Me (Q1–Q3)	7 (5.0–11.0)	7 (5.0–10.0)	0.532	7 (5.0–12.0)	7 (5.0–10.0)	0.695
Barthel Scale, Me (Q1–Q3)	30 (10.0–57.5)	25 (10.0–52.5)	0.620	68 (10.0–100.0)	25 (10.0–40.0)	<0.001
ADL Scale, Me (Q1–Q3)	3 (1.0–5.0)	4 (1.0–6.0)	0.513	5 (2.0–6.0)	2 (1.0–5.0)	<0.001
Frail NH Scale, Me (Q1–Q3)	6 (3.0–8.0)	6 (3.0–7.0)	0.188	3 (1.0–7.0)	6 (4.0–8.0)	<0.001
Comorbidities n (%)						
Hypertension	108 (79.4)	88 (71.0)	0.114	58 (75.3)	126 (75.0)	0.956
Ischemic heart disease	52 (38.2)	46 (37.7)	0.930	22 (29.0)	70 (41.9)	0.053
Ischemic heart disease with a history of a myocardial infraction	13 (9.6)	8 (6.6)	0.389	5 (6.6)	17 (10.2)	0.358
Atrial fibrillation	39 (28.7)	26 (21.0)	0.152	11 (14.3)	44 (26.2)	0.038
Heart failure	54 (40.0)	55 (44.7)	0.415	19 (25.0)	82 (48.8)	<0.001
Storke	37 (27.2)	30 (24.2)	0.579	13 (16.9)	47 (28.0)	0.061
Frailty	53 (41.7)	61 (49.2)	0.235	24 (32.4)	86 (51.8)	0.005
Parkinson’s disease	21 (15.4)	20 (16.1)	0.879	8 (10.4)	31 (18.5)	0.109
Epilepsy	12 (8.8)	11 (8.9)	0.989	8 (10.4)	17 (10.1)	0.948
Dementia	70 (51.9)	48 (38.7)	0.034	29 (38.7)	81 (48.2)	0.167
Depression	54 (39.7)	48 (39.0)	0.911	22 (28.6)	76 (45.5)	0.012
Diabetes	43 (31.6)	40 (32.3)	0.912	20 (26.0)	59 (35.1)	0.155
Thyroid diseases	28 (20.6)	22 (17.7)	0.561	12 (15.6)	33 (19.6)	0.446
Hypercholesterolemia	53 (39.3)	38 (31.9)	0.224	32 (43.8)	58 (34.7)	0.180
COPD/asthma	9 (6.6)	15 (12.3)	0.117	7 (9.3)	17 (10.1)	0.850
Pulmonary fibrosis	2 (1.5)	9 (7.5)	0.018	4 (5.3)	7 (4.2)	0.701
Gastric and duodenal ulcer disease	21 (15.4)	18 (14.6)	0.856	8 (10.5)	30 (17.9)	0.144
Liver cirrhosis	6 (4.4)	5 (4.1)	0.890	3 (4.0)	7 (4.2)	0.936
CKD	31 (22.8)	24 (19.5)	0.519	11 (14.5)	38 (22.6)	0.141
RUTI	31 (22.8)	23 (18.7)	0.418	14 (18.4)	38 (22.6)	0.458
Osteoarthritis	73 (53.7)	72 (58.1)	0.477	29 (37.7)	107 (63.7)	<0.001
Rheumatoid arthritis	3 (2.4)	6 (4.4)	0.387	2 (2.6)	6 (3.6)	0.703
Oncology diseases	13 (9.6)	18 (14.6)	0.217	13 (16.9)	18 (10.8)	0.189

Legend: Q1–Q3—25th and 75th percentiles; CKD—chronic kidney disease; COPD—chronic obstructive pulmonary disease; Me—median; n—number; RUTI—recurrent urinary tract infections; * After excluding data on 15 persons due to lack of data.

## Data Availability

The datasets generated and analyzed during the current study are not publicly available but are available from the corresponding author upon reasonable request.

## Abbreviations

LTCFs—long-term care facilities; PPSV23—23-valent polysaccharide vaccine; PCV13—13-valent polysaccharide vaccine; PCV10—10-valent polysaccharide vaccine; ADL—the activities of daily living score; CAP—community-acquired pneumonia.
